# Different Patterns of Mental Health Outcomes among Road Traffic Crash Survivors: A Prospective Cohort Study

**DOI:** 10.3390/ijerph18041564

**Published:** 2021-02-07

**Authors:** Jelena Kovacevic, Ivica Fotez, Ivan Miskulin, Davor Lesic, Maja Miskulin, Terezija Berlancic, Ivan Vukoja, Slavko Candrlic, Hrvoje Palenkic, Marija Candrlic

**Affiliations:** 1 Faculty of Medicine Osijek, Josip Juraj Strossmayer University of Osijek, 31000 Osijek, Croatia; dr.kovacevic.jelena@gmail.com (J.K.); ivan.miskulin@mefos.hr (I.M.); tberlancic@gmail.com (T.B.); iv.vukoja@gmail.com (I.V.); hrpal@net.hr (H.P.); 2 Faculty of Dental Medicine and Health Osijek, Josip Juraj Strossmayer University of Osijek, 31000 Osijek, Croatia; ivica.fotez@vt.t-com.hr (I.F.); davor.lesic@fdmz.hr (D.L.); slavko.candrlic@fdmz.hr (S.C.); marija.candrlic@fdmz.hr (M.C.)

**Keywords:** anxiety, depression, injury, injury severity, posttraumatic stress disorder, road traffic crash, Croatia

## Abstract

This study aimed to investigate factors associated with the symptoms of mental disorders following a road traffic crash (RTC). A prospective cohort of 200 people was followed for 6 months after experiencing an RTC. The cohort was comprised of uninjured survivors and injured victims with all levels of road traffic injury (RTI) severity. Multivariable logistic regression analyses were performed to evaluate the associations between the symptoms of depression, posttraumatic stress disorder and anxiety one and six months after the RTC, along with sociodemographic factors, health status before and after the RTC, factors related to the RTI and factors related to the RTC. The results showed associations of depression, anxiety, and posttraumatic stress disorder symptoms with sociodemographic factors, factors related to the health status before and after the RTC and factors related to the RTC. Factors related to the RTI showed associations only with depression and posttraumatic stress disorder symptoms. Identifying factors associated with mental disorders following an RTC is essential for establishing screening of vulnerable individuals at risk of poor mental health outcomes after an RTC. All RTC survivors, regardless of their RTI status, should be screened for factors associated with mental disorders in order to successfully prevent them.

## 1. Introduction

Although the World Health Organization (WHO) considers road traffic crashes (RTCs) to be preventable, nearly 3700 people die in RTCs worldwide every day [1], and for every death there are at least 20 people that sustain non-fatal injuries [2]. In the Republic of Croatia, there were 9695 RTCs with reported casualties in 2019, where 297 people died and 12,885 were injured [3]. 

Since RTCs are one of the leading causes of premature death in the world, the United Nations General Assembly proclaimed “The decade of action for road safety 2011–2020” aiming to save lives by ensuring road safety, vehicle safety, improving road-user behavior and post-crash response [4]. Nevertheless, RTCs are still the leading cause of death for children and young adults [1], while road traffic crash (RTC) survivors suffer a wide range of consequences, e.g., functional impairments, cognitive dysfunctions, psychological suffering and poor quality of life [5]. 

A significant proportion of RTC victims develop psychological disorders [6,7,8,9,10,11,12,13,14,15,16,17,18,19,20,21], most commonly posttraumatic stress disorder (PTSD), depressive disorder, driving phobia and other anxiety disorders [22,23]. A recent meta-analysis determined a pooled prevalence of PTSD following an RTC of 22.3%, with disparities among studies due to measuring instruments, country, gender, race and education level [18]. Prevalence of depressive disorder following a RTC ranges from 7.8% to 63% [8,10,11,13,14,16,17,21,24,25], while prevalence of anxiety disorder ranges from 19.4% to 60% [11,24]. Consistent predictors of PTSD following an RTC are lack of social support, perceived threat to life, fatal outcomes in the RTC, acute stress disorder, previous physical and emotional problems and compensation claim [26], while influence of road traffic injury (RTI) severity on PTSD showed contradicting results demanding more research [5,26]. Predictive factors determined for depression and anxiety following an RTC are perceived life-threat [27], poor pre-RTC health status, female gender [28], and RTI severity [29], but literature data are not as abundant as for PTSD. 

A recent meta-analysis concluded that psychological stress following an RTC is significant, but it was not clear whether it was caused by RTI or traumatic event itself, and suggested future research including uninjured controls [19]. So far, there have been no prospective studies of RTC outcomes that included uninjured RTC survivors. Prospective studies of RTC victims and mental health outcomes or its predictors have never been conducted in the Croatian population. Therefore, the aim of this study was to prospectively follow RTC survivors that had recently experienced an RTC with or without RTI in order to determine mental health outcomes and its predictors in this population. Different patterns of mental health outcomes may serve as guidelines for designing institutional response to this matter. 

## 2. Materials and Methods

**Figure 1:**
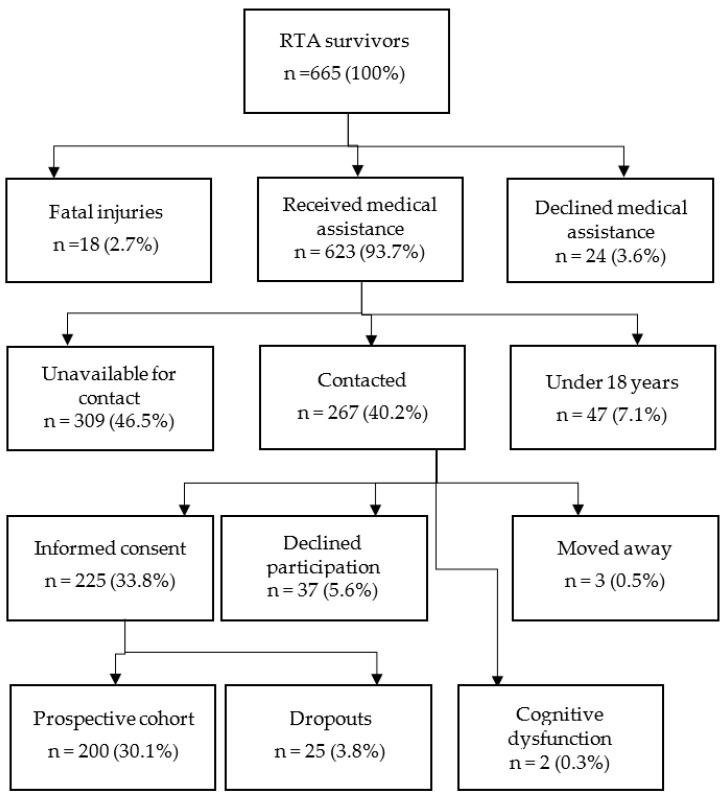
Participant recruitment process.

A prospective cohort was followed between December 2016 and September 2017. The research was conducted at the Institute of emergency medicine of Vukovar-Srijem County in Croatia and it was approved by the Ethics Committee of the Faculty of Medicine Osijek, Croatia (Ethical Approval Code: 2158-61-07-17-211). A cohort of 200 RTC survivors was followed during six months after experiencing an RTC. Participants gave informed consent for participation in the study. Inclusion criteria were recent RTC experience and ≥18 years of age. Exclusion criteria were minor age and cognitive and mental health problems resulting in inability to give consent and provide necessary information. At one month and six months after an RTC, the participants gave information regarding their psychological and physical health status, socioeconomic status, compensation status, RTC characteristics, road traffic injuries (RTIs) and pre-RTC health status. Cohort recruitment is presented in Figure 1.

Sociodemographic characteristics explored were age, sex, place of residence, education level, employment status, marital status, self-perceived economic status and religiousness. Pre-RTC health status included smoking habit, alcohol consumption, psychoactive substance use, body weight and height, presence of chronic physical and psychiatric diseases, medication use, previous traumatic or RTC experience and permanent pain. RTC characteristics included road user type, total number of crashed vehicles, total number of (RTIs) and road traffic fatalities (RTFs), unconsciousness in the RTC, post-RTC amnesia, fault for perpetrating the RTC, compensation claim and obtained compensation. RTI characteristics explored were injury status, injury severity, self-perceived life-threat, pain after the RTI, hospitalization and duration of hospitalization, surgical treatment, and rehabilitation after the RTC. Post-RTC health status explored whether there was another traumatic event or RTC in the follow-up period, new chronic diseases, sick leave duration, work status, invalidity, retirement due to RTC, driving phobia, permanent pain after the RTC, location and frequency of pain, pain management, medication use, smoking, alcohol and psychoactive substance consumption, subjective feeling of recovery and perception of general health. Body mass index (BMI) was calculated from self-reported body height and weight according to WHO [30]. Presence of PTSD symptoms was assessed using the PTSD Check List—Civilian Version (PCL-C) [31]. Depression symptoms were assessed using a Beck Depression Inventory—version I (BDI) [32] and anxiety symptoms were assessed using a Beck Anxiety Inventory (BAI) [33]. Abbreviated Injury Scale [34] and New Injury Severity Scale [35] were used to assess RTA injury severity. NISS classifies multiple injuries as mild, moderate, serious, severe and critical. Critical, severe, and serious injuries were analyzed as one category.

The normality of data distributions was checked by the Kolmogorov–Smirnov test. Descriptive statistics were used to describe the socio-demographic characteristics of study participants and RTC details, as well as the characteristics of the participants and their mental health outcomes 1 month and 6 months following an RTC. Multiple logistic regression was used to explore factors associated with depression, anxiety, and PTSD symptoms 1 month and 6 months following an RTC, i.e., six prognostic models were proposed. The associations between explored risk factors and mentioned mental health outcomes of RTC in each model were presented as odds ratios (ORs) with a 95% confidence intervals and *p*-values. To make models reliable and select the factors that have an impact on the output, backward elimination was used with a selection criterion of 0.157 because such selection criterion is emphasized as the most appropriate for prognostic models [36]. Data analysis was performed by SPSS statistical software package version 22.0 (SPSS Inc., Chicago, IL, USA). The statistical significance level was set at *p* < 0.05.

## 3. Results

### 3.1. Characteristics of the Cohort

The cohort comprised 200 participants with median age of 42.5 years (interquartile range 28.3–56.0) and 54% were males. There were 48.5% of participants in the younger age group (18 to 41 years) and 51.5% of participants in the older age group (over 41 years). Urban residence was reported by 43.5% and rural residence was reported by 56.5% of the participants. Primary education was reported by 19.0%, secondary education was reported by 62.5% and university education was reported by 18.5% of the participants. Unemployment was reported by 26.0% of the participants, while 58.0% were employed and 16.0% were retired. Being single was reported by 35.5% of the participants, while 64.5% were in a relationship. Under average self-perceived economic status was reported by 20.0% of the participants, while 58.0% reported average and 22.0% reported above average self-perceived economic status. Religious believes was reported by 90.5% of the participants. According to BMI, 3.5% of the participants were underweight, 37.0% had normal weight, 38.5% were overweight and 21.0% were obese. Smoking habit was reported by 35.5% of the participants, while alcohol consumption was reported by 50.5% of the participants. Only 3.5% of the participants reported psychoactive substance use. Medication use was reported by 51.0% of the participants. Psychiatric medications were used by 3.5%, non-psychiatric medications were used by 39.0%, and both types of medications were used by 8.5% of the participants. RTC experience in the past was reported by 42.0% of the participants, while 52.0% reported previous traumatic experience. Previously, diagnosed PTSD was reported by 3.5% of the participants, previous chronic illness was reported by 42.0% and previous psychiatric illness was reported by 11.0% of the participants. Permanent pain before the RTC was reported by 9.5% of the participants. The non-participants were of similar age, sex and primary injury characteristics as the participants.

RTC details of the participants are presented in Table 1.

### 3.2. One-Month Follow-Up

Post-RTC characteristics of the participants one month following an RTC are presented in Table 2.

Mental health outcomes were assessed one month after the RTC experience. Symptoms of PTSD were reported by 35.5%, depression symptoms were reported by 20.0% and anxiety symptoms were reported by 12.0% of the participants. Comorbidity of investigated mental health outcomes was present in 22.5% of the RTC survivors: 2.5% had symptoms of both anxiety and depression, 15.5% reported symptoms of depression and PTSD, while 3% reported symptoms of anxiety and PTSD. Symptoms of all three investigated psychological disorders were reported by 1.5% of the participants. Mental health outcomes of RTC survivors one month following the RTC are presented in Table 3. 

The multivariable regression model for depression symptoms at 1-month follow-up showed that depression symptoms were significantly more likely to be present in irreligious participants (OR = 9.625, 95% CI = 2.419–38.297, *p* = 0.001), in those who used medications before the RTC (OR = 0.249, 95% CI = 0.099–0.629, *p* = 0.003), and in participants with self-perceived life-threat (OR = 0.255, 95% CI = 0.099–0.660, *p* = 0.005). The model also showed that depression symptoms were significantly less likely to be present in participants with mild RTI in comparison to those with critical, severe, or serious RTI (OR = 0.160, 95% CI = 0.053–0.480, *p* = 0.001), in drivers in comparison to cyclists/pedestrians (OR = 0.156, 95% CI = 0.039–0.627, *p* = 0.009), and in co-drivers/passengers in comparison to cyclists/pedestrians (OR = 0.175, 95% CI = 0.041–0.742, *p* = 0.018) (Table 4). Finally, the model revealed that there were no statistically significant associations between depression symptoms at 1-month follow-up and the age group of study participants, their self-perceived economic status, existence of chronic disease before the RTC, type of medications used before the RTC, existence of pain after the RTC, hospitalization due to the RTC, duration of hospitalization due to the RTC, surgical treatment due to the RTC, unconsciousness in the RTC and amnesia from the RTC.

The multivariable regression model for anxiety symptoms at 1-month follow-up showed that anxiety symptoms were significantly less likely to be present in males (OR = 0.065, 95% CI = 0.006–0.682, *p* = 0.023), in participants who did not use psychoactive substances before the RTC (OR = 0.001, 95% CI = 0.000–0.097, *p* = 0.002), in those who did not have psychiatric disease before the RTC (OR = 0.086, 95% CI = 0.011–0.644, *p* = 0.017) and those who did not suffer from permanent pain before the RTC (OR = 0.035, 95% CI = 0.005–0.247, *p* = 0.001) (Table 5). The model also revealed that there were no statistically significant associations between anxiety symptoms at 1-month follow-up and existence of chronic disease before the RTC, type of medications used before the RTC, self-perceived life-threat, existence of pain after the RTC, rehabilitation due to the RTC and road user type. 

The multivariable regression model for PTSD symptoms at 1-month follow-up showed that PTSD symptoms were significantly more likely to be present in participants without past experience of the RTC (OR = 2.453, 95% CI = 1.107–5.435, *p* = 0.027), in those who were not hospitalized after the RTC (OR = 5.697, 95% CI = 1.240–26.173, *p* = 0.025) and in participants hospitalized from 4 to 10 days in comparison to those who were hospitalized for 11 or more days (OR = 7.647, 95% CI = 1.519–38.510, *p* = 0.014). The model also showed that PTSD symptoms were significantly less likely to be present in participants without psychiatric disease before the RTC (OR = 0.201, 95% CI = 0.063–0.641, *p* = 0.007), in participants who did not use medications before the RTC (OR = 0.436, 95% CI = 0.203–0.935, *p* = 0.033), in participants who did not sustain the RTI (OR = 0.049, 95% CI = 0.008–0.288, *p* = 0.001), in participants with mild RTI in comparison to those with serious, severe or critical RTI (OR = 0.152, 95% CI = 0.046–0.504, *p* = 0.002), in participants without self-perceived life-threat (OR = 0.297, 95% CI = 0.140–0.631, *p* = 0.002) and in those who had not claimed compensation after the RTC (OR = 0.355, 95% CI = 0.165–0.763, *p* = 0.008) (Table 6). Finally, the model revealed that there were no statistically significant associations between PTSD symptoms at 1-month follow-up and participants’ sex, employment status, self-perceived economic status, alcohol consumption before the RTC, existence of chronic disease before the RTC, type of medications used before the RTC, existence of pain after the RTC, and rehabilitation due to the RTC.

### 3.3. Six-Month Follow-Up

Post-RTC characteristics of RTC survivors six months following the RTC are presented in Table 7.

Mental health outcomes 6 months following the RTC experience showed reduction in the number of participants with the symptoms of psychological disorders. Symptoms of PTSD were reported by 20.5% of RTC survivors. Depression symptoms were present in 13.5% and anxiety symptoms were present in 3.5% of RTC victims. Comorbidity of mental health disorders was present in 18.0% of the participants. Comorbid anxiety and depression symptoms were present in 2.5%, PTSD and depression symptoms were present in 11.0%, while PTSD and anxiety symptoms were present in 2.5% of the RTC survivors. Symptoms of all three investigated mental disorders were present in 2% of the participants. Mental health outcomes of RTC victims six months following the RTC are presented in Table 8.

The multivariable regression model for depression symptoms at 6-month follow-up showed that depression symptoms were significantly less likely to be present in participants who did not experience repeated RTC (OR = 0.020, 95% CI = 0.003–0.150, *p* < 0.001), in participants who did not suffer permanent pain after the RTC (OR = 0.067, 95% CI = 0.010–0.436, *p* = 0.005), in participants with permanent pain level after the RTC between 1 and 3 in comparison to those with permanent pain level after the RTC between 7 and 10 (OR = 0.032, 95% CI = 0.002–0.543, *p* = 0.017), in participants who did not increase alcohol consumption after the RTC (OR = 0.011, 95% CI = 0.000–0.327, *p* = 0.009), in participants who felt completely recovered after the RTC in comparison to those who reported deterioration of health status after the RTC (OR = 0.054, 95% CI = 0.004–0.731, *p* = 0.028) and in participants who partially recovered after the RTC in comparison to those who reported deterioration of health status after the RTC (OR = 0.020, 95% CI = 0.001–0.348, *p* = 0.007) (Table 9). Finally, the model revealed that there were no statistically significant associations between depression symptoms at 6-month follow-up and the age group of study participants, their religiousness, existence of past traumatic experience (before the RTC), existence of chronic disease before the RTC, existence of psychiatric disease before the RTC, use of medications before the RTC, type of medications used before the RTC, RTI severity, self-perceived life-threat, road user type, new chronic disease after the RTC, duration of sick-leave after the RTC, existence of driving phobia after the RTC, another trauma after the RTC, presence of pain after the RTC, increase in pain level after the RTC, increase in medication use after the RTC and presence of any RTC consequence. 

The multivariable regression model for anxiety symptoms at 6-month follow-up showed that anxiety symptoms were significantly less likely to be present in a driver of a motor vehicle in comparison to a cyclist/pedestrian (OR = 0.098, 95% CI = 0.011–0.874, *p* = 0.037) and in participants who did not increase medication use after the RTC (OR = 0.140, 95% CI = 0.022–0.898, *p* = 0.038) (Table 10). The model also revealed that there were no statistically significant associations between anxiety symptoms at 6-month follow-up and the age group of study participants, their employment status, alcohol consumption before the RTC, existence of chronic disease before the RTC, existence of permanent pain before the RTC, new chronic disease after the RTC, increase in pain level after the RTC, permanent pain level after the RTC, and presence of any RTC consequence. 

The multivariable regression model for PTSD symptoms at 6-month follow-up showed that PTSD symptoms were significantly more likely to be present in participants who were not religious (OR = 7.554, 95% CI = 2.059–27.721, *p* = 0.002). The model also showed that PTSD symptoms were significantly less likely to be present in participants who did not claim compensation (OR = 0.368, 95% CI = 0.142–0.951, *p* = 0.039), in a driver of a motor vehicle in comparison to a cyclist/pedestrian (OR = 0.125, 95% CI = 0.030–0.512, *p* = 0.004), in a co-driver/passenger in a motor vehicle in comparison to a cyclist/pedestrian (OR = 0.063, 95% CI = 0.013–0.313, *p* = 0.001), in participants who did not suffer permanent pain after the RTC (OR = 0.189, 95% CI = 0.068–0.528, *p* = 0.001), and in participants who did not increase medication use after the RTC (OR = 0.191, 95% CI = 0.071–0.513, *p* = 0.001) (Table 11). Finally, the model revealed that there were no statistically significant associations between PTSD symptoms at 6-month follow-up and the age group of study participants, alcohol consumption before the RTC, past traumatic experience (before the RTC), existence of PTSD before the RTC, existence of chronic disease before the RTC, existence of psychiatric disease before the RTC, existence of permanent pain before the RTC, use of medications before the RTC, type of medications used before the RTC, experience of RTI, RTI severity, self-perceived life-threat, existence of pain after the RTC, surgery after the RTC, duration of sick leave after the RTC, change of a job due to the RTC, driving phobia after the RTC, presence of pain after the RTC, increase in pain level after the RTC, permanent pain level after the RTC, increase in smoking after the RTC, presence of any RTC consequence, and perception of health after the RTC. 

## 4. Discussion

The study prospectively followed uninjured RTC survivors and injured RTC victims with all levels of injury severity for six months following the RTC, unlike other prospective studies of RTC victims that only included injured RTC survivors. Outcomes on physical and psychological health were assessed one month and six months following the RTC experience. 

A full recovery after a six-month follow-up was reported by 59.5% of RTC survivors, while other research has reported this for 46.7% of recovered RTC victims two years following the RTC [37]. This study reported only 5.5% of RTC survivors on a sick leave for longer than 6 months, while other studies have obtained higher rates of sick leave even two years after an RTC [38]. Differences can be explained by different structure of injury severity among participants of different studies since research showed association between the recovery after an RTC and injury severity [5].

Pain frequency decreased in the RTC survivors during the follow-up, but even after six months, 21.0% of RTC victims suffered permanent pain, as opposed to 9.5% of the participants that suffered permanent pain before the RTC. Study results showed that one in five RTC survivors suffered chronic pain, which is a significant number of people that experience an RTC every year. Public health importance of persistent pain in development of disability and mental disorders, such as depression and PTSD, is well established [39]. 

One month following an RTC, 40.5% of the participants suffered symptoms of an investigated psychological disorder, while six months after an RTC, 23.5% of all RTC survivors reported symptoms of an investigated mental health disorder. Other studies found one half of RTC victims to be suffering from mental disorders 12 to 24 months after an RTC [11,40]. It is considered that one in four RTC survivors suffer from psychological consequences up to one year after the RTC [21,41]. 

The prospective cohort was characterized by a high prevalence of PTSD and depression symptoms and a low prevalence of anxiety symptoms during the research period. The obtained prevalence results were within the expected range, and are similar to other studies of mental health outcomes in RTC survivors [5,7,9,10,11,12,13,14,15,16,17,18,21,25,26,40,42,43,44,45]. Comorbidity of mental health outcomes determined in this study has also been established in other studies of RTC victims [8,11,13,15,16,17,25,40]. RTC survivors with comorbid mental disorders should be the focus of attention, since research found comorbidity to be the predictor of poorer mental health outcomes in the long term [11]. During the prospective follow-up, 9.5% of RTC victims developed driving phobia, which is similar to other research investigating fear of driving that developed in 9% of survivors of RTCs in Serbia [17].

The study showed an association between mental disorders and sociodemographic factors, as well as health status in terms of the RTC, RTI and RTC characteristics. The significance of certain factors changed during the follow-up period. Study results showed that socioeconomic factors were not significantly associated with mental health outcomes of RTCs. Other research into RTCs also showed that socioeconomic factors such as employment status or education level showed no association with depression or anxiety symptoms [46,47]. Regression models for one-month follow-up found irreligiousness to be a risk factor for depression symptoms, while at six-month follow-up, irreligiousness was determined to be a risk factor for PTSD symptoms, but not for depression symptoms. Other studies of RTC survivors and RTC outcomes did not explore religiousness, but this author’s preliminary studies showed similar results [47]. In general, religiousness is a well-known factor influencing mental health [48,49].

Results showed that female sex was a risk factor for anxiety symptoms at one-month follow-up. Although a few studies found no association between gender and mental health of RTC victims [26,40,50], there are far more studies that have found an association between female gender and mental health disorders in RTC survivors [9,13,15,17,18,28,44,47,51,52]. 

Health status before the RTC, including permanent pain before the RTC, previous psychiatric disease, previous RTC experience, previous psychoactive substance use and medication use, showed an association with mental health problems during the follow-up period. Similar to other research, the study results showed that poor physical and mental health before the RTC was a risk factor for developing psychological disorders after the RTC experience [9,11,26,28,40,43,50,53].

Regression models found factors related to RTI, such as a sustaining a RTI, RTI severity, self-perceived life-threat, hospitalization and its duration, to be associated with depression and PTSD symptoms, while symptoms of anxiety showed no significant association with the RTI. Other studies also found RTI [5,9,17,20,21,40,45,47,54], hospitalization [17,47], pain [9,13,47,55] and life-threat [8,20,27,47] to be associated with poor mental health outcomes, such as PTSD and/or depression. Anxiety symptoms in RTC survivors unrelated to RTI was also reported in earlier research [47]. 

Compensation claims were found to be associated with PTSD symptoms of RTC victims. Compensation processes following RTC are a well-known predictor of PTSD in the literature [9,26,56]. It is thought that the constant reminders of the RTC and traumatic details during the compensation process have negative effects on RTC victims with PTSD symptoms [26]. Others have found an association between PTSD and driving phobia, while the regression model in this study found no association between these [57].

Regression models have shown that vulnerable road users, i.e., pedestrians and cyclists, had a higher risk of developing all of the investigated mental health disorders during the prospective follow-up in comparison with motor-vehicle drivers and passengers/co-drivers. Other research of RTC survivors from Europe and India that included several road user types in the study also identified vulnerable road users as those being at risk of psychological disorders after the RTC [21,45]. It is possible that this vulnerability to mental disorders results from the RTI, since all pedestrians and cyclists in this study reported RTIs.

The study results found an association between symptoms of mental disorders and post-RTC health status six months after the RTC. Regression models identified repeated RTC, permanent pain following an RTC, level of permanent pain, increase of alcohol and medication use and exacerbation of health status to be risk factors for mental health problems. Other research showed anxiety following the RTC to be negative prognostic factor associated with permanent pain and disability [58]. High levels of pain have already been associated with the development of chronic pain and mental and physical disability; therefore, early management of pain and comorbidities such as PTSD, depression and anxiety can reduce development of chronic pain and related disabilities [39].

Literature data has found an association between PTSD and depression in RTC victims [13,15,17,59]. This study determined factors associated with symptoms of PTSD and depression to be similar and largely related to pre-RTC and post-RTC health status and RTI. This may serve as a direction for the future research and for a development of screenings and interventions targeting RTC victims with risk factors. Screening might be set in healthcare facilities, such as trauma wards and rehabilitation centers, where injured RTC victims would be easily reachable for screening [47]. Early interventions are important, since research has shown that RTC survivors with PTSD have greater risk of developing other mental disorders in the long term [11]. The study results showed that anxiety symptoms following the RTC are associated with poor pre-RTC health status, and not with RTI, which has also been established previously [47]. 

Recent systematic reviews of the most important factors of poor recovery following the RTC included high levels of pain, duration and intensity of pain, physical and mental health status before the RTC, PTSD, RTI severity and compensation procedure [39,60,61], which is congruent with the results of this study. This study indicated some unexplored factors that deserve more attention such as religiousness as a protective factor and medication use as a negative prognostic factor. Preliminary research by this author also showed pre-RTC medication use to be a significant factor associated with mental health outcomes of RTC victims [47].

### Strengths and Limitations

The limitations of this study included the use of self-reported data, rather than using medical records for detecting pre-existing medical conditions. Participants represented only 31.3% of all RTC survivors, mostly due to lack of contact information. The response rate of 84.2% was high among those RTC victims who were contacted. Despite limitations, the study has several strengths. The study was set up prospectively, and a high number of variables were explored. To ensure systematic approach to RTI, uninjured RTC survivors and injured RTC victims with all types of RTI severity were included in the study, unlike some studies that have only included hospitalized RTC victims [10,12,14,21,45,56]. RTC survivors were engaged outside compensation settings to avoid possible secondary gain of the participants.

## 5. Conclusions

Consistent predictors of poor mental health outcomes in RTC survivors should be the foundation for creating effective screening tools used to determine vulnerable RTC survivors at risk of developing psychological consequences following the RTC. Such RTC victims should be provided with psychological support and other interventions, such as effective pain management to prevent development of mental disorders following the RTC. 

## Figures and Tables

**Table 1:** RTC details of the participants.

Characteristics	N	%
**Road user type**		
Driver of a motor vehicle	122	61.0
Co-driver/passenger	61	30.5
Cyclist/pedestrian	17	8.5
**Total number of motor vehicles in the RTC**		
0	1	0.5
1	92	46.0
<1	107	53.5
**Total number of injured people**		
0	29	14.5
1	84	42.0
2 to 3 people	72	36.0
4 and more	15	7.5
**RTFs**		
No	195	97.5
Yes	5	2.5
**Fault for perpetrating the RTC**		
No	123	61.5
Yes	70	35.0
Unestablished	7	3.5
**Compensation claim**		
No	113	56.5
Yes	87	43.5
**Obtained compensation**		
No	180	90.0
Yes	20	10.0

**Table 2:** Characteristics of the participants 1 month following an RTC.

Characteristics	N	%
**Number of RTIs**		
None	31	15.5
One	45	22.5
Multiple	124	62.0
**Location of the RTI**		
None	31	15.5
Head	18	9.0
Face	2	1.0
Neck	8	4.0
Chest	8	4.0
Abdomen	1	0.5
Spine	3	1.5
Hands	3	1.5
Legs	10	5.0
Multiple	116	58.0
**Primary RTI**		
None	31	15.5
Head	58	29.0
Neck	37	18.5
Chest	19	9.5
Abdomen	12	6.0
Hands	17	8.5
Legs	26	13.0
**RTI severity**		
None	31	15.5
Mild	96	48.0
Moderate	36	18.0
Serious	28	14.0
Severe	6	3.0
Critical	3	1.5
**Self-perceived life-threat in the RTC**		
No	108	54.0
Yes	92	46.0
**Unconsciousness from the RTC**		
No	168	84.0
Yes	32	16.0
**Amnesia following the RTC**		
No	172	86.0
Yes	28	14.0
**Days with post- RTC amnesia**		
No amnesia	172	86.0
1 to 9	7	3.5
10 to 30	4	2.0
<30	17	8.5
**Hospitalization**		
No	136	68.0
Yes	64	32.0
**Duration of hospitalization**		
None	136	68.0
1 to 3	27	13.5
4 to 10	19	9.5
<10	18	9.0
**Surgery**		
No	180	90.0
Yes	20	10.0
**Rehabilitation treatment**		
No	154	77.0
Yes	46	23.0
**Location of the pain**		
No pain	47	23.5
Head	11	5.5
Face	1	0.5
Neck	11	5.5
Spine	5	2.5
Chest	10	5.0
Abdomen	1	0.5
Upper extremities	12	6.0
Lower extremities	12	6.0
Multiple locations	90	45.0
**Pain frequency**		
Never	47	23.5
Circumstantial	35	17.5
Occasional	62	31.0
Permanent	56	28.0

**Table 3:** Mental health outcomes at 1-month follow up.

Mental Health Outcomes	N	%
**Symptoms of PTSD**		
No	129	64.5
Yes	71	35.5
**Symptoms of depression**		
Normal mood	160	80.0
Mild mood disturbance	24	12.0
Borderline clinical depression	3	1.5
Moderate depression	11	5.5
Severe depression	2	1.0
**Symptoms of anxiety**		
Low anxiety	191	95.5
Moderate anxiety	8	4.0
Severe anxiety	1	0.5

**Table 4:** The multivariable regression model for depression symptoms at 1-month follow-up.

Variables	OR	OR 95% CI	*p*
**Religiousness**			
Yes	Reference		
No	9.625	2.419–38.297	0.001 **
**Use of medications before RTC**			
Yes	Reference		
No	0.249	0.099–0.629	0.003 **
**RTI severity**			
Serious, severe, or critical	Reference		
Moderate	0.393	0.117–1.326	0.132
Mild	0.160	0.053–0.480	0.001 **
None	0.535	0.124–2.316	0.403
**Self-perceived life-threat**			
Yes	Reference		
No	0.255	0.099–0.660	0.005 **
**Road user type**			
Cyclist/pedestrian	Reference		
Co-driver/passenger	0.175	0.041–0.742	0.018 *
Driver of a motor vehicle	0.156	0.039–0.627	0.009 **

OR—odds ratio, 95% CI—95% confidence interval, * *p* < 0.05, ** *p* < 0.01.

**Table 5:** The multivariable regression model for anxiety symptoms at 1-month follow-up.

Variables	OR	OR 95% CI	*p*
**Sex**			
Female	Reference		
Male	0.065	0.006–0.682	0.023 *
**Use of psychoactive substances before the RTC**			
Yes	Reference		
No	0.001	0.000–0.097	0.002 **
**Psychiatric disease before the RTC**			
Yes	Reference		
No	0.086	0.011–0.644	0.017 *
**Permanent pain before the RTC**			
Yes	Reference		
No	0.035	0.005–0.247	0.001 **
**Self-perceived life-threat**			
Yes	Reference		
No	0.107	0.011–1.078	0.058

OR—odds ratio, 95% CI—95% confidence interval, * *p* < 0.05, ** *p* < 0.01.

**Table 6:** The multivariable regression model for PTSD symptoms at 1-month follow-up.

Variables	OR	OR 95% CI	*p*
Sex			
Female	Reference		
Male	0.850	0.397–1.821	0.676
**Past experience of the RTC**			
Yes	Reference		
No	2.453	1.107–5.435	0.027 *
**Psychiatric disease before the RTC**			
Yes	Reference		
No	0.201	0.063–0.641	0.007 **
**Use of medications before the RTC**			
Yes	Reference		
No	0.436	0.203–0.935	0.033 *
**RTI**			
Yes	Reference		
No	0.049	0.008–0.288	0.001 **
**RTI severity**			
Serious, severe, or critical	Reference		
Moderate	0.417	0.129–1.343	0.143
Mild	0.152	0.046–0.504	0.002 **
**Self-perceived life-threat**			
Yes	Reference		
No	0.297	0.140–0.631	0.002 **
**Hospitalization after the RTC**			
Yes	Reference		
No	5.697	1.240–26.173	0.025 *
**Duration of hospitalization**			
11 or more days	Reference		
4 to 10 days	7.647	1.519–38.510	0.014 *
1 to 3 days	2.823	0.600–13.277	0.189
**Compensation claim**			
Yes	Reference		
No	0.355	0.165–0.763	0.008 **

OR—odds ratio, 95% CI—95% confidence interval, * *p* < 0.05, ** *p* < 0.01.

**Table 7:** Characteristics of the participants at 6-month follow-up.

Characteristics	N	%
**Repeated RTC**		
No	191	95.5
Yes	9	4.5
**Another traumatic experience**		
No	178	89.0
Yes	22	11.0
**New chronic disease**		
No	188	94.0
Yes	12	6.0
**Type of new chronic disease**		
None	188	94.0
Hypertension	1	0.5
Cardiac disease	1	0.5
Lung disease	1	0.5
Carcinoma	1	0.5
Psychiatric disease	1	0.5
Other	7	3.5
**Duration of sick leave following the RTC**		
No sick leave	100	50.0
<1 month	40	20.0
1–3 months	43	21.5
4–6 months	6	3.0
>6 months	11	5.5
**Change of a job due to the RTC**		
No	194	97.0
Yes	6	3.0
**Less working hours due to the RTC**		
No	198	99.0
Yes	2	1.0
**Retirement due to the RTC**		
No	199	99.5
Yes	1	0.5
**Invalidity due to the RTC**		
No	198	99.0
Yes	2	1.0
**Driving phobia**		
No	181	90.5
Yes	19	9.5
**Pain frequency following the RTC**		
Never	105	52.5
Circumstantial	40	20.0
Occasional	34	17.0
Permanent	21	10.5
**Pain location following the RTC**		
No pain	105	52.5
Head	8	4.0
Face	1	0.5
Neck	6	3.0
Spine	7	3.5
Chest	4	2.0
Abdomen	1	0.5
Hands	13	6.5
Legs	13	6.5
Multiple body parts	42	21.0
**Pain management**		
None	92	46.0
Medication	51	25.5
Rehabilitation treatment	12	6.0
Other	2	1.0
Combination of treatments	43	21.5
**Permanent pain following the RTC**		
No	158	79.0
Yes	42	21.0
**Level of permanent pain**		
No pain	158	79.0
1–3	14	7.0
4–6	13	6.5
7–10	15	7.5
**Increase of pain level**		
No	133	66.5
Yes	67	33.5
**Increase of medication use**		
No	149	74.5
Yes	51	25.5
**Increase of alcohol consumption**		
No	197	98.5
Yes	3	1.5
**Increase of smoking**		
No	193	96.5
Yes	7	3.5
**Increase of psychoactive substance use**		
No	199	99.5
Yes	1	0.5
**Presence of any RTC consequence**		
No	107	53.5
Yes	93	46.5
**Perception of health**		
Complete recovery	119	59.5
Partial recovery	53	26.5
Steady state	21	10.5
Exacerbation	7	3.5

**Table 8:** Mental health outcomes at 6-month follow-up.

Mental Health Outcomes	N	%
**PTSD symptoms**		
No	159	79.5
Yes	41	20.5
**Depression**		
Normal mood	173	86.5
Mild mood disturbance	11	5.5
Borderline clinical depression	7	3.5
Moderate depression	8	4.0
Severe depression	1	0.5
**Anxiety**		
Low anxiety	193	96.5
Moderate anxiety	6	3.0
Severe anxiety	1	0.5

**Table 9:** The multivariable regression model for depression symptoms at 6-month follow-up.

Variables	OR	OR 95% CI	*p*
**Religiousness**			
Yes	Reference		
No	3.274	0.672–15.951	0.142
**Chronic disease before the RTC**			
Yes	Reference		
No	0.229	0.038–1.375	0.107
**Use of medications before the RTC**			
Yes	Reference		
No	0.182	0.019–1.713	0.136
**Type of medications used before the RTC**			
Various chronic diseases therapy (including psychiatric therapy)	Reference		
Psychiatric therapy	2.554	0.130–50.248	0.537
Various chronic diseases therapy	0.205	0.031–1.350	0.099
**Chronic disease after the RTC**			
Yes	Reference		
No	0.101	0.009–1.101	0.060
**Repeated RTC**			
Yes	Reference		
No	0.020	0.003–0.150	<0.001 **
**Permanent pain after the RTC**			
Yes	Reference		
No	0.067	0.010–0.436	0.005 **
**Permanent pain level after the RTC**			
Level 7–10	Reference		
Level 4–6	0.118	0.010–1.416	0.092
Level 1–3	0.032	0.002–0.543	0.017 *
**Increase in alcohol consumption after the RTC**			
Yes	Reference		
No	0.011	0.000–0.327	0.009 **
**Perception of health after the RTC**			
Deterioration	Reference		
Stable	1.474	0.135–16.058	0.750
Partial recovery	0.020	0.001–0.348	0.007 **
Complete recovery	0.054	0.004–0.731	0.028 *

OR—odds ratio, 95% CI—95% confidence interval, * *p* < 0.05, ** *p* < 0.01.

**Table 10:** The multivariable regression model for anxiety symptoms at 6-month follow-up.

Variables	OR	OR 95% CI	*p*
**Chronic disease before the RTC**			
Yes	Reference		
No	0.223	0.022–2.225	0.201
**Road user type**			
Cyclist/pedestrian	Reference		
Co-driver/passenger	0.122	0.014–1.033	0.054
Driver of a motor vehicle	0.098	0.011–0.874	0.037 *
**New chronic disease after the RTC**			
Yes	Reference		
No	0.166	0.023–1.193	0.074
**Increase in medication use after the RTC**			
Yes	Reference		
No	0.140	0.022–0.898	0.038 *

OR—odds ratio, 95% CI—95% confidence interval, * *p* < 0.05.

**Table 11:** The multivariable regression model for PTSD symptoms at 6-month follow-up.

Variables	OR	OR 95% CI	*p*
**Religiousness**			
Yes	Reference		
No	7.554	2.059–27.721	0.002 **
**Psychiatric disease before the RTC**			
Yes	Reference		
No	0.312	0.080–1.206	0.091
**Use of medications before the RTC**			
Yes	Reference		
No	0.255	0.054–1.193	0.083
**Type of medications used before the RTC**			
Various chronic diseases therapy (including psychiatric therapy)	Reference		
Psychiatric therapy	0.962	0.117–7.940	0.972
Various chronic diseases therapy	0.213	0.045–1.000	0.050
**Compensation claim**			
Yes	Reference		
No	0.368	0.142–0.951	0.039 *
**Road user type**			
Cyclist/pedestrian	Reference		
Co-driver/passenger	0.063	0.013–0.313	0.001 **
Driver of a motor vehicle	0.125	0.030–0.512	0.004 **
**New chronic diseases after the RTC**			
Yes	Reference		
No	0.200	0.037–1.069	0.060
**Permanent pain after the RTC**			
Yes	Reference		
No	0.189	0.068–0.528	0.001 **
**Increase in medications use after the RTC**			
Yes	Reference		
No	0.191	0.071–0.513	0.001 **

OR—odds ratio, 95% CI—95% confidence interval, * *p* < 0.05, ** *p* < 0.01.

## References

[1] World Health Organization (2018). Global Status Report on Road Safety 2018.

[2] World Health Organization (2015). Global Status Report on Road Safety 2015.

[3] Croatian Bureau of Statistics (2019). Registered Road Vehicles and Road Traffic Accidents in 2018.

[4] United Nations Road Safety Collaboration Decade of Action for Road Safety 2011–2020 Seeks to Save Millions of Lives. https://www.who.int/roadsafety/decade_of_action/en/.

[5] Hours M., Chossegros L., Charnay P., Tardy H., Nhac-Vu H.T., Boisson D., Luauté J., Laumon B. (2013). Outcomes one year after a road accident: Results from the ESPARR cohort. Accid. Anal. Prev..

[6] Mayou R., Bryant B., Ehlers A. (2001). Prediction of psychological outcomes one year after a motor vehicle accident. Am. J. Psychiatry.

[7] Merecz-Kot D., Waszkowska M., Wężyk A. (2015). Mental health status of drivers--Motor vehicle accidents perpetrators. Med. Pr..

[8] Guest R., Tran Y., Gopinath B., Cameron I.D., Craig A. (2018). Prevalence and psychometric screening for the detection of major depressive disorder and post-traumatic stress disorder in adults injured in a motor vehicle crash who are engaged in compensation. BMC Psychol..

[9] Chossegros L., Hours M., Charnay P., Bernard M., Fort E., Boisson D., Sancho P.O., Yao S.N., Laumon B. (2011). Predictive factors of chronic post-traumatic stress disorder 6 months after a road traffic accident. Accident Anal. Prev..

[10] Copanitsanou P., Drakoutos E., Kechagias V. (2018). Posttraumatic stress, depressive emotions, and satisfaction with life after a road traffic accident. Otrhop. Nurs..

[11] Kenardy J., Edmed S.L., Shourie S., Warren J., Crothers A., Brown E.A., Cameron C.M., Heron-Delaney M. (2018). Changing patterns in the prevalence of posttraumatic stress disorder, major depressive episode and generalized anxiety disorder over 24 months following a road traffic crash: Results from the UQ SuPPORT study. J. Affect. Disord..

[12] Ning L., Guan S., Liu J. (2017). Impact of personality and social support on posttraumatic stress disorder after traffic accidents. Medicine.

[13] Khodadadi-Hassankiadeh N., Nayeri N.D., Shahsavari H., Yousefzadeh-Chabok S., Haghani H. (2017). Predictors of post-traumatic stress disorder among victims of serious motor vehicle accidents. Int. J. Community Based Nurs. Midwifery.

[14] Hruska B., Irish L.A., Pacella M.L., Sledjeski E.M., Delahanty D.L. (2014). PTSD symptom severity and psychiatric comorbidity in recent motor vehicle accident victims: A latent class analysis. J. Anxiety Disord..

[15] Yohannes K., Gebeyehu A., Adera T., Ayano G., Fekadu W. (2018). Prevalence and correlates of post-traumatic stress disorder among survivors of road traffic accidents in Ethiopia. Int. J. Ment. Health Syst..

[16] Asuquo J.E., Edet B.E., Abang I.E., Essien E.A., Osakwe O.G., Aigbomain E.J., Chigbundu K.C. (2017). Depression and posttraumatic stress disorder among road traffic accident victims managed in a Tertiary hospital in Southern Nigera. Niger. J. Clin. Pract..

[17] Dickov A., Martinović-Mitrović S., Vučković N., Siladji-Mladenović D., Mitrović D., Jovičević M., Mišić-Pavkov G. (2009). Psychiatric consequences of stress after a vehicle accident. Psychiat. Danub..

[18] Lin W., Gong L., Xia M., Dai W. (2018). Prevalence of posttraumatic stress disorder among road traffic accident survivors. A PRISMA compliant meta-analysis. Medicine.

[19] Craig A., Tran Y., Guest R., Gopinath B., Jagnoor J., Bryant R., Collie A., Tate R., Kenardy J., Middleton J.W. (2016). Psychological impact of injuries sustained in motor vehicle crashes: Systematic review and meta-analysis. BMJ Open.

[20] Craig A., Elbers N.A., Jagnoor J., Gopinath B., Kifley A., Dinh M., Pozzato I., Ivers R.Q., Nicholas M., Cameron I.D. (2017). The psychological impact of traffic injuries sustained in a road crash by bicyclists: A prospective study. Traffic Inj. Prev..

[21] Papadakaki M., Ferraro O.E., Orsi C., Otte D., Tzamalouka G., von-der-Geest M., Lajunen T., Ozkan T., Morandi A., Sarris M. (2017). Psychological distress and physical disability in patients sustaining severe injuries in road traffic crashes: Results from a one-year cohort study from three European countries. Injury.

[22] Mayou R., Bryant B. (2002). Outcome 3 years after a road traffic accident. Psychol. Med..

[23] Bryant R.A., O’Donnell M.L., Creamer M., McFarlane A.C., Clark C.R., Silove D. (2010). The psychiatric sequelae of traumatic injury. Am. J. Psychiatry.

[24] Littleton S.M., Hughes D.C., Poustie S.J., Robinson B.J., Neeman T., Smith P.N., Cameron I.D. (2012). The influence of fault on health in the immediate post-crash period following road traffic crashes. Injury.

[25] Ehring T., Ehlers A., Gluksman E. (2008). Do cognitive models help in predicting the severity of posttraumatic stress disorder, phobia, and depression after motor vehicle accidents? A prospective longitudinal study. J. Consult. Clin. Psychol..

[26] Heron-Delaney M., Kenardy J., Charlton E., Matsuoka Y. (2013). A systematic review of predictors of posttraumatic stress disorder (PTSD) for adult road traffic crash survivors. Injury.

[27] Jagnoor J., De Wolf A., Nicholas M., Maher C.G., Casey P., Blyth F., Harris I.A., Cameron I.D. (2015). Restriction in functioning and quality of life is common in people 2 months after compensable motor vehicle crashes: Prospective cohort study. Inj. Epidemiol..

[28] Elbers N.A., Akkermans A.J., Lockwood K., Craig A., Cameron I.D. (2015). Factors that challenge health for people involved in the compensation process following a motor vehicle crash: A longitudinal study. BMC Public Health.

[29] Balayan K., Kahloon M., Tobia G., Postolova A., Peek H., Akopyan A., Lord M., Brownstein A., Aziz A., Nwabueze U. (2014). The Impact of Posttraumatic Stress Disorder on the Quality of Life: A Systematic Review. Int. Neuropsychiatr. Dis. J..

[30] World Health Organization Regional Office for Europe. Body Mass Index..

[31] Weathers F.W., Litz B.T., Herman D., Huska J., Keane T. (1994). The PTSD Checklist—Civilian Version (PCL-C).

[32] Beck A.T., Steer R.A., Garbin M.G. (1988). Psychometric properties of the Beck Depression Inventory: Twenty-five years of evaluation. Clin. Psychol. Rev..

[33] Beck A.T., Epstein N., Brown G., Steer R.A. (1988). An inventory for measuring clinical anxiety: Psychometric properties. J. Consult. Clin. Psych..

[34] Civil I.D., Schwab C.W. (1988). The Abbreviated Injury Scale, 1985 Revision: A Condensed Chart for Clinical Use. J. Traum..

[35] Stevenson M., Segui-Gomez M., Lescohier I., Di Scala C., McDonald-Smith G. (2001). An overview of the injury severity score and the new injury severity score. Inj. Prev..

[36] Heinze G., Dunkler D. (2017). Five myths about variable selection. Transpl. Int..

[37] Tournier C., Charnay P., Tardy H., Chossegros L., Carnis L., Hours M. (2014). A few seconds to have an accident, a long time to recover: Consequences for road accident victims from the ESPARR cohort 2 years after the accident. Accid. Anal. Prev..

[38] Heron-Delaney M., Warren J., Kenardy J.A. (2017). Predictors of non-return to work 2 years’ post-injury in road traffic crash survivors: Results from the UQ SuPPORT study. Injury.

[39] Samoborec S., Ruseckaite R., Ayton D., Evans S. (2018). Biopsychosocial factors associated with non-recovery after a minor transport-related injury: A systematic review. PLoS ONE.

[40] Cartwright A. (2017). The Psychological Effects of Road Traffic Accidents (RTAs): An Exploration of a United Kingdom Medico-Legal Examiner’s Career of RTA Assessments. Psychiatr. Psychol. Law.

[41] Ścigała D.K., Zdankiewicz-Ścigała E. (2019). The Role in Road Traffic Accident and Anxiety as Moderators Attention Biases in Modified Emotional Stroop Test. Front. Psychol..

[42] Yaşan A., Guzel A., Tamam Y., Ozkan M. (2009). Predictive factors for acute stress disorder and posttraumatic stress disorder after motor vehicle accidents. Psychopathology.

[43] Ursano R.J., Fullerton C.S., Epstein R.S., Crowley B., Kao T.C., Vance K., Craig K.J., Dougall A.L., Baum A. (1999). Acute and chronic posttraumatic stress disorder in motor vehicle accident victims. Am. J. Psychiatry.

[44] Iteke O., Bakare M.O., Agomoh A.O., Uwakwe R., Onwukwe J.U. (2011). Road traffic accidents and posttraumatic stress disorder in an orthopedic setting in South-Eastern Nigeria: A controlled study. Scand. J. Trauma Resusc. Emerg. Med..

[45] Seethalakshmi R., Dhavale H.S., Gawande S., Dewan M. (2006). Psychiatric morbidity following motor vehicle crashes: A pilot study from India. J. Psychiatr. Pract..

[46] Littleton S.M., Cameron I.D., Poustie S.J., Hughes D.C., Robinson B.J., Neeman T., Smith P.N. (2011). The association of compensation on longer term health status for people with musculosceletal injuries following road traffic crashes: Emergency department inception cohort study. Injury.

[47] Kovacevic J., Miskulin M., Degmecic D., Vcev A., Leovic D., Sisljagic V., Simic I., Palenkic H., Vcev I., Miskulin I. (2020). Predictors of mental health outcomes in road traffic accident survivors. J. Clin. Med..

[48] Moreira-Almeida A., Neto F.L., Koenig H.G. (2006). Religiousness and mental health: A review. Braz. J. Psychiatry.

[49] Vitorino L.M., Lucchetti G., Leão F.C., Vallada H., Peres M.F.P. (2018). The association between spirituality and religiousness and mental health. Sci. Rep..

[50] Fekadu W., Mekonen T., Belete H., Belete A., Yohannes K. (2019). Incidence of Post-Traumatic Stress Disorder After Road Traffic Accident. Front. Psychiatry.

[51] Hasselberg M., Kirsebom M., Bäckström J., Berg H.Y., Rissanen R. (2019). I did NOT feel like this at all before the accident: Do men and women report different health and life consequences of a road traffic injury?. Inj. Prev..

[52] Giummarra M.J., Black O., Smith P., Collie A., Hassani-Mahmooei B., Arnold C.A., Gong J., Gabbe B.J. (2018). A population-based study of treated mental health and persistent pain conditions after transport injury. Injury.

[53] Stein D.J., Karam E.G., Shahly V., Hill E.D., King A., Petukhova M., Atwoli L., Bromet E.J., Florescu S., Haro J.M. (2016). Post-traumatic stress disorder associated with life-threatening motor vehicle collisions in the WHO World Mental Health Surveys. BMC Psychiatry.

[54] Hours M., Bernard M., Charnay P., Chossegros L., Javouhey E., Fort E., Boisson D., Sancho P.O., Laumon B. (2010). Functional outcome after road-crash injury: Description of the ESPARR victims cohort and 6-month follow-up results. Accid. Anal. Prev..

[55] Ravn S.L., Hartvigsen J., Hansen M., Sterling M., Andresen T.E. (2018). Do post-traumatic pain and post-traumatic stress symptomatology mutually maintain each other? A systematic review of cross-lagged studies. Pain.

[56] Murgatroyd D.F., Harris I.A., Tran Y., Cameron I.D. (2016). The association between seeking financial compensation and injury recovery following motor vehicle related orthopaedic trauma. BMC Musculoskel. Dis..

[57] Mairean C. (2020). Posttraumatic stress symptoms, fear and avoidance of driving, and aberrant driving behaviors. The moderating role of gender. J. Transp. Health.

[58] Littleton S.M., Hughes D.C., Poustie S.J., Robinson B.J., Neeman T., Smith P.N., Cameron I.D. (2014). An early intervention programme had no detectable influence on the health status of people with musculoskeletal injuries following road traffic crashes: Comparative study. Injury.

[59] Angerpointner K., Weber S., Tschech K., Schubert H., Herbst T., Ernstberger A., Kerschbaum M. (2020). Posttraumatic stress disorder after minor trauma—A prospective cohort study. Med. Hypotheses.

[60] Sarrami P., Armstrong E., Naylor J.M., Harris I.A. (2017). Factors predicting outcome in whiplash injury: A systematic meta-review of prognostic factors. J. Orthop. Traumatol..

[61] Smits E., Brakenridge C., Gane E., Warren J., Heron-Delaney M., Kenardy J., Johnston V. (2019). Identifying risk of poor physical and mental health recovery following a road traffic crash: An industry-specific screening tool. Accid. Anal. Prev..

